# LncRNA OIP5‐AS1 Modulates the Biological Behaviour of Lung Cancer Cells by Regulating the hsa‐miR‐29b‐3p/ZIC5 Axis

**DOI:** 10.1111/jcmm.70596

**Published:** 2025-05-08

**Authors:** Long Liang, Ying Luo, Danyang Li, Yongchang Sun

**Affiliations:** ^1^ Department of Respiratory and Critical Care Medicine Peking University Third Hospital Beijing China

**Keywords:** hsa‐miR‐29b‐3p, lung adenocarcinoma, OIP5‐AS1, ZIC5

## Abstract

Existing knowledge regarding the involvement of lncRNA OIP5‐AS1 in lung adenocarcinoma (LUAD) development is still incomplete and requires further investigation. Our research aimed to reveal the function of OIP5‐AS1 in LUAD. We evaluated the level of OIP5‐AS1 and its association with clinicopathological factors in LUAD. The research examined the potential implications of targeting OIP5‐AS1 in mitigating the invasive properties of lung cancer cells. A nude mouse xenograft model was utilised to examine tumour growth. We used bioinformatics data and a dual‐luciferase reporter assay to study the interactions between OIP5‐AS1 and hsa‐miR‐29b‐3p. OIP5‐AS1 was significantly overexpressed in LUAD, with a higher level correlating with adverse clinicopathological features. Knockdown of OIP5‐AS1 resulted in notable decreases in LUAD cell growth, movement, and aggressive behaviour, accompanied by a decrease in tumour size in vivo. Furthermore, OIP5‐AS1 was confirmed to act as a molecular sponge for hsa‐miR‐29b‐3p. The elevated expression of hsa‐miR‐29b‐3p intensified the inhibitory outcomes of OIP5‐AS1 knockdown on LUAD cell properties. ZIC5 was experimentally determined to be a direct molecular target of hs‐miR‐29b‐3p, emphasising its integral position in the regulatory interaction. This study reveals a new regulatory route involving OIP5‐AS1, hsa‐miR‐29b‐3p and ZIC5 in LUAD pathogenesis. Given its oncogenic traits, OIP5‐AS1 presents a promising predictive biomarker and therapeutic target for optimising lung cancer treatment.

## 1 Introduction

Lung cancer remains one of the most prevalent and deadly cancers worldwide, with approximately 2 million new cases and 1.76 million deaths annually [1]. Its high incidence is strongly linked to environmental factors, particularly smoking, air pollution and workplace exposure to harmful substances. While current treatments such as surgery [2], chemotherapy [3], radiotherapy [4] and targeted therapies [5] offer some strategies, challenges persist due to tumour heterogeneity, complex biological behaviours and high rates of recurrence and metastasis. Early diagnosis and timely intervention are therefore crucial to improving survival rates and patient outcomes.

As a member of the long non‐coding RNA (lncRNA) family, OIP5‐AS1 demonstrates essential involvement in various physiological events, notably impacting oncogenic cell proliferation and metastatic spread [6]. Some studies show that OIP5‐AS1 is overexpressed in ovarian [7] and pancreas [8], driving tumour development. Elevated levels of OIP5‐AS1 are often tied to poor clinical outcomes, suggesting its crucial participation in oncogenesis. However, studies investigating the role of OIP5‐AS1 in lung cancer, particularly lung adenocarcinoma (LUAD), remain limited. This gap highlights the novelty and significance of our research, which focuses on understanding the involvement of OIP5‐AS1 in LUAD.

It is well established that lncRNAs often regulate tumour progression by modulating downstream miRNAs. Through database analysis, we identified hsa‐miR‐29b‐3p as a downstream target of OIP5‐AS1. hsa‐miR‐29b‐3p is typically downregulated in cancerous tissues. This downregulation correlates with enhanced cancer cell proliferation, migration and invasion [9]. Through the precise modulation of oncogenesis‐related genetic elements, hsa‐miR‐29b‐3p exerts a significant influence on the restructuring of the tumour milieu while simultaneously fine‐tuning immune regulatory pathways. It specifically targets and reduces ZIC5 level, an important transcription factor in lung cancer progression [10]. Consequently, the expression level of hsa‐miR‐29b‐3p may significantly influence lung cancer progression. The present study has thoroughly examined the interaction mechanisms between OIP5‐AS1, hsa‐miR‐29b‐3p and ZIC5. Our findings have illuminated the role of OIP5‐AS1 in the multifaceted pathogenesis of LUAD, offering new perspectives for understanding this disease.

## 2 Materials and Methods

### 2.1 lnCAR Database

lnCAR (https://lncar.renlab.org/) is a comprehensive database that integrates gene annotations from Ensembl and RefSeq, providing lncRNA expression profiles based on over 57,000 cancer samples. By re‐annotating microarray data, it reveals differential expression and prognosis information in human cancers. lnCAR includes expression analysis, survival analysis, co‐expression analysis, KEGG pathway enrichment and ceRNA analysis, facilitating the exploration of lncRNA functions.

### 2.2 GEPIA Database

GEPIA (Gene Expression Profiling Interactive Analysis, http://gepia.cancer‐pku.cn/) is an online tool used to analyse gene expression in cancer and normal tissues. It integrates large datasets from the TCGA (The Cancer Genome Atlas) and GTEx (Genotype‐Tissue Expression) projects, allowing users to perform interactive data queries and visualisations.

### 2.3 TCGA Samples

TCGA (https://portal.gdc.cancer.gov/) currently encompasses 36 distinct types of cancer, including breast cancer, lung cancer, colorectal cancer and glioblastoma. The TCGA initiative integrates a variety of data types, including genomic data, transcriptomic data, epigenetic data and clinical data.

### 2.4 Starbase

Starbase (http://starbase.sysu.edu.cn/) is widely utilised in cancer research, especially for forecasting miRNA targets, examining cancer gene regulatory networks and discovering new RNA biomarkers. Researchers can use Starbase to predict miRNA or lncRNA targets, investigate their regulatory pathways and potentially discover new therapeutic targets.

### 2.5 OncomiR

OncomiR (https://oncomir.org/) compiles a comprehensive collection of miRNA data linked to various cancers, including not just expression profiles, but also target genes and relevant clinical information. OncomiR utilises extensive data to facilitate in‐depth survival analysis, enabling users to evaluate the correlation between specific miRNA levels and survival outcomes.

### 2.6 UALCAN Platform

UALCAN (https://ualcan.path.uab.edu/) is an easily accessible and intuitive web platform designed to support the analysis of cancer omics datasets. This approach enables the contrasting of transcriptional profiles between malignant and normal tissues, while simultaneously investigating correlations between genetic markers and patient overall survival (OS), as well as evaluating clinical parameters such as disease stage and demographic features.

### 2.7 Cultivation of Cells and Quantitative Real‐Time PCR

Cell lines including human bronchial epithelial cells (HBE) and lung adenocarcinoma lines (A549 and PC9) were acquired from the department of respiratory medicine at Peking University Third Hospital and cultured in RPMI 1640 medium supplemented with 10% FBS. cDNA was synthesised with the Evo M‐MLV Reverse Transcription Premix Kit (Cat. AG11728, China) for RNA and the cDNA Synthesis Kit for miRNAs (Cat. NRT0004, China). Relative gene expression was quantified using the SYBR Green Pro Tag HS Premixed qPCR Kit I (Cat. AG11739, China), and expression levels were calculated by the 2^−ΔΔ*Ct*^ method. Primer sequences are detailed in Table S1.

### 2.8 Transfection Assay

Three small interfering RNAs (siRNAs) designed to target OIP5‐AS1, as well as a negative control siRNA (siNC), were synthesised by Beijing Tsingke Biotechnology Co. Ltd. The specific sequences of these siRNAs are provided in Table S2. Taking advantage of the high‐efficiency transfection capabilities of Lipofectamine 3000 (Invitrogen), we successfully delivered siRNAs into A549 and PC9 cells to establish stable knockdown models. Prior to cellular transfection, a complex was formulated through the integration of siRNAs with the miR‐29b‐3p mimic, which was subsequently co‐delivered into the cells as a combined reagent system.

### 2.9 CCK‐8 and EdU Staining

Cells were seeded in 96‐well plates at a density of 5 × 10^3^ cells per well and incubated at 37°C with 5% CO_2_ until adherent. At 0, 24, 48 and 72 h, 10 μL of CCK‐8 solution was added to each well and incubated for 1 h at 37°C. Absorbance at 450 nm was measured using a microplate reader to assess cell viability and proliferation. Each experiment was performed in triplicate, and data were presented as the mean ± standard deviation (SD). For cell proliferation, the BeyoClick EdU‐594 Cell Proliferation Kit was used. Cells were fixed with 4% paraformaldehyde, incubated with the reaction solution for 30 min, and stained with Hoechst 33342. Fluorescence images were captured using a Leica microscope and analysed with IMAGEJ software.

### 2.10 Cell Migration and Invasion

We employed transwell chambers featuring 8 μm pores for conducting the cell migration and invasion experiments. We seeded 2 × 10^4^ cells into the upper compartment of a dual‐chamber system, where they were maintained in a serum‐free medium under controlled conditions. Simultaneously, in the lower chamber, we placed a medium rich in 10% FBS, acting as a robust chemoattractant to stimulate cellular migration. Following a 24‐h incubation period, the cells were fixed, subjected to crystal violet staining, and subsequently quantified in three arbitrarily selected fields of view under a light microscope. For the invasion assay, Matrigel (GoldenGene, Beijing, China) was applied to the chamber membrane.

### 2.11 Luciferase Reporter Assay

Our study focused on deciphering its complete regulatory network, with specific attention to elucidating mechanisms whereby this miRNA regulates transcriptional activity through direct engagement with the 3′UTRs of OIP5‐AS1 and ZIC5. To validate these predicted regulatory relationships, we amplified the hsa‐miR‐29b‐3p recognition motifs within the 3′UTRs of both genes using PCR, generating constructs for both wild‐type (WT) and mutant (MUT) sequences. Using the pmirGLO dual‐luciferase reporter system, we generated four plasmid variants: OIP5‐AS1 (WT and MUT) and ZIC5 (WT and MUT). The 293T cells were co‐transfected with these plasmids along with either an hsa‐miR‐29b‐3p mimic or a negative control miRNA (miR‐NC). Subsequently, following a 48‐h incubation period, luciferase activity was quantified utilising a multifunctional plate reader (Synergy LX, manufactured by BioTek in the USA), with Renilla luciferase serving as a normalisation factor.

### 2.12 Immunohistochemical Staining Procedure

Tissue sections were deparaffinised, rehydrated and subjected to antigen retrieval in sodium citrate buffer (pH 6.0) at 95°C for 15 min. Endogenous peroxidase activity was blocked with 3% hydrogen peroxide for 10 min. After blocking with 5% BSA, sections were incubated overnight at 4°C with anti‐ZIC5 antibody (1:100, ARP33669_P050, USA), followed by secondary antibody for 1 h at room temperature. DAB was used for visualisation, and haematoxylin for counterstaining. Sections were dehydrated, mounted and analysed under a light microscope.

### 2.13 Tumorigenicity Assay in Nude Mice

The nude mice (6 weeks old, female) tumorigenesis assay was provided by the Third Hospital of Peking University. Different gene constructs were transfected into the A549 cell line, including siNC, siOIP5‐AS1‐2 and hsa‐miR‐29b‐3p + siOIP5‐AS1‐2 groups. Following transfection, 1–2 × 10^6^ A549 cells from each group were collected, resuspended in 0.1 mL of PBS, and injected into both sides of the abdomen of each nude mouse. After a period of 1 month, the dimensions of the tumour, specifically its length (*L*) and width (*W*), were recorded. Subsequently, the tumour volume (*V*) was calculated employing the formula: *V* = (*L* × *W* ^2^)/2. After euthanising the mice, the tumours were excised, weighed and analysed. Measurement of OIP5‐AS1, hsa‐miR‐29b‐3p and ZIC5 expression in cancer tissues delineated their tumorigenesis‐linked transcriptional patterns.

### 2.14 Statistical Analysis

Clinical data from TCGA were analysed using R software (version 4.2.3). The Chi‐square test and paired *t*‐test were used to analyse the statistical data during the experiment. Data visualisation was done with GraphPad Prism software (version 9), with a *p*‐value < 0.05 considered statistically significant.

## 3 Results

### 3.1 Elevated OIP5‐AS1 Expression in Lung Cancer Associated With Clinicopathological Features

**FIGURE 1:**
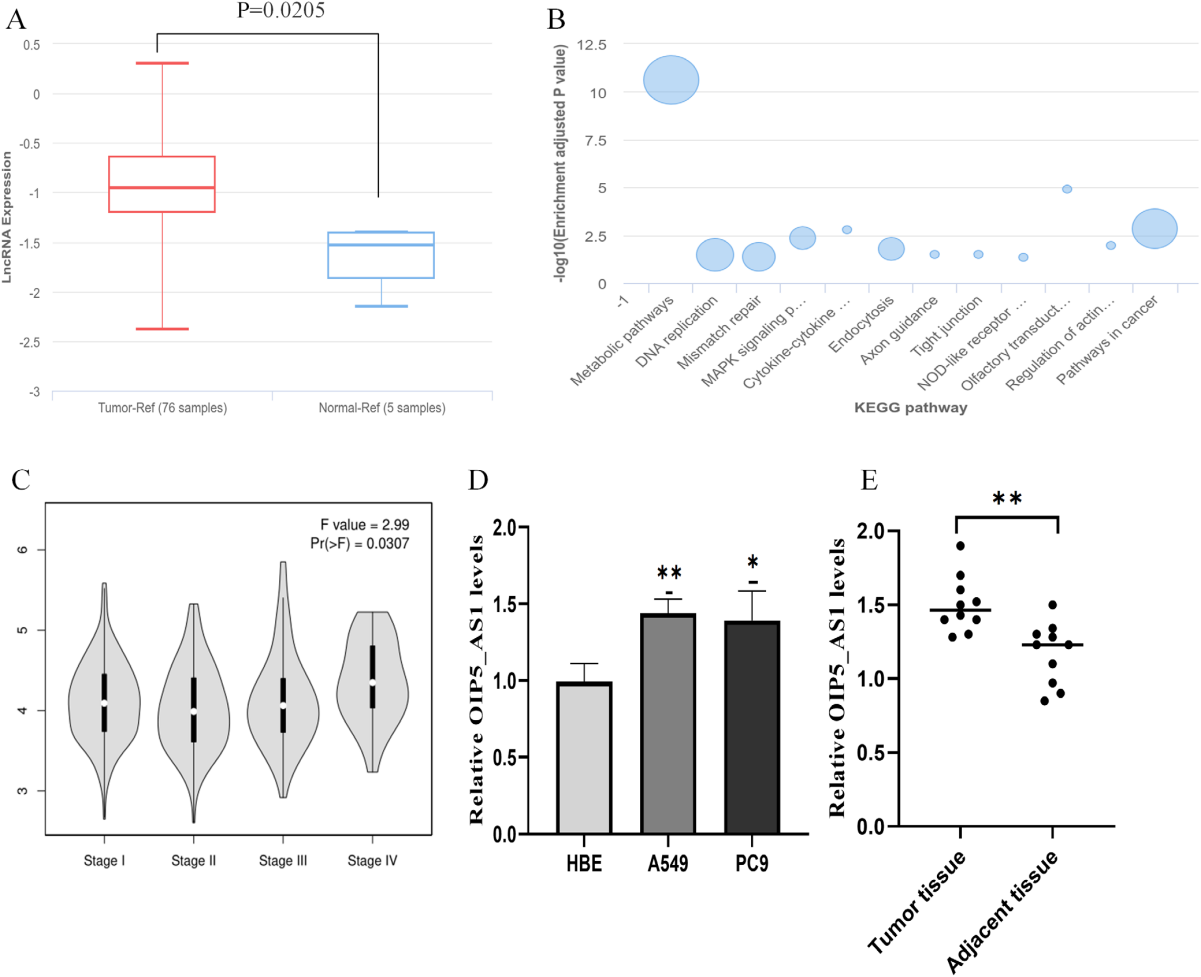
Relative expression of lncRNA OIP5‐AS1 in lung cancer tissues and cell lines. (A) The lnCAR database revealed significant differential expression of the lncRNA OIP5‐AS1 between 76 tumour tissues and 5 normal tissues (*p* = 0.0205). (B) KEGG signalling pathways associated with lncRNA OIP5‐AS1. (C) Differential expression of lncRNA OIP5‐AS1 across different stages of LUAD, analysed using the GEPIA platform. (D) A comparison was conducted to evaluate lncRNA OIP5‐AS1 in normal airway epithelium (HBE) versus lung cancer cells (A549 and PC9). (E) Comparison of OIP5‐AS1 expression levels in lung cancer tissues and adjacent normal tissues (*n* = 10). Three independent experiments were conducted. Data are presented as mean ± SD, based on at least triplicate experiments. Statistical significance is indicated as follows: *p < 0.05; **p < 0.01. LUAD, lung adenocarcinoma.

Our initial data mining from the lnCAR repository disclosed a statistically significant elevation of lncRNA OIP5‐AS1 in LUAD specimens compared to normal controls (Tumour samples = 76, normal samples = 5, *p* = 0.0205, Figure 1A). KEGG annotation classified OIP5‐AS1 into three functional categories: (i) metabolic pathways, (ii) genome maintenance systems (DNA replication/mismatch repair) and (iii) cancer regulatory networks, with the tumour‐associated pathways being most relevant to our research focus (Figure 1B). We observed a marked increase in OIP5‐AS1 expression in advanced LUAD, especially in stage IV (*p* = 0.0307, Figure 1C). We downloaded 541 LUAD cases from the TCGA database. After removing missing values, we retained 364 complete data points for clinical parameter analysis. The results indicated a notable correlation between the varied expression of OIP5‐AS1 and clinical grade (*p* = 0.010), as well as a significant link with lymph node metastasis (*p* = 0.002), as outlined in Table 1. We conducted a comparative transcriptomic analysis and found that OIP5‐AS1 was overexpressed in A549 and PC9 cell lines compared to normal respiratory epithelium (*p* < 0.05, see Figure 1D). A similar trend was observed in lung cancer tissues (*p* < 0.05, see Figure 1E).

**TABLE 1:** Correlations between LncRNA OIP5‐AS1 expression and clinicopathological characteristics in LUAD patients (*n* = 364).

Patient characteristics	Cases (*n* = 364)	Expression of OIP5‐AS1		*p* (chi‐square test)
		Low (*n* = 91)	High (*n* = 273)	
Age (years)				0.952
≤ 65	167	42	125	
> 65	197	49	148	
Gender				0.276
Male	182	50	132	
Female	182	41	141	
Race				0.489
American	1	0	1	
Asian	6	3	3	
Black	29	9	20	
White	285	67	218	
Not reported	43	12	31	
Clinical grade				0.010*
Stage I–II	280	61	219	
Stage III–IV	84	30	54	
Pathologic T				0.535
T I–II	315	77	238	
T III–IV	49	14	35	
Pathologic N				0.002**
N0	234	46	188	
N1–3	130	45	85	
Pathologic M				0.800
M0	342	86	256	
M1	22	5	17	

*Note:* Low expression of OIP5‐AS1: the expression of OIP5‐AS1 was lower than the quartile OIP5‐AS1; High expression of OIP5‐AS1: the expression of OIP5‐AS1 was higher than the quartile OIP5‐AS1.

* *p* < 0.05.

** *p* < 0.01.

### 3.2 Down‐Regulation of OIP5‐AS1 Affects the Biological Processes of LUAD Cells

**FIGURE 2:**
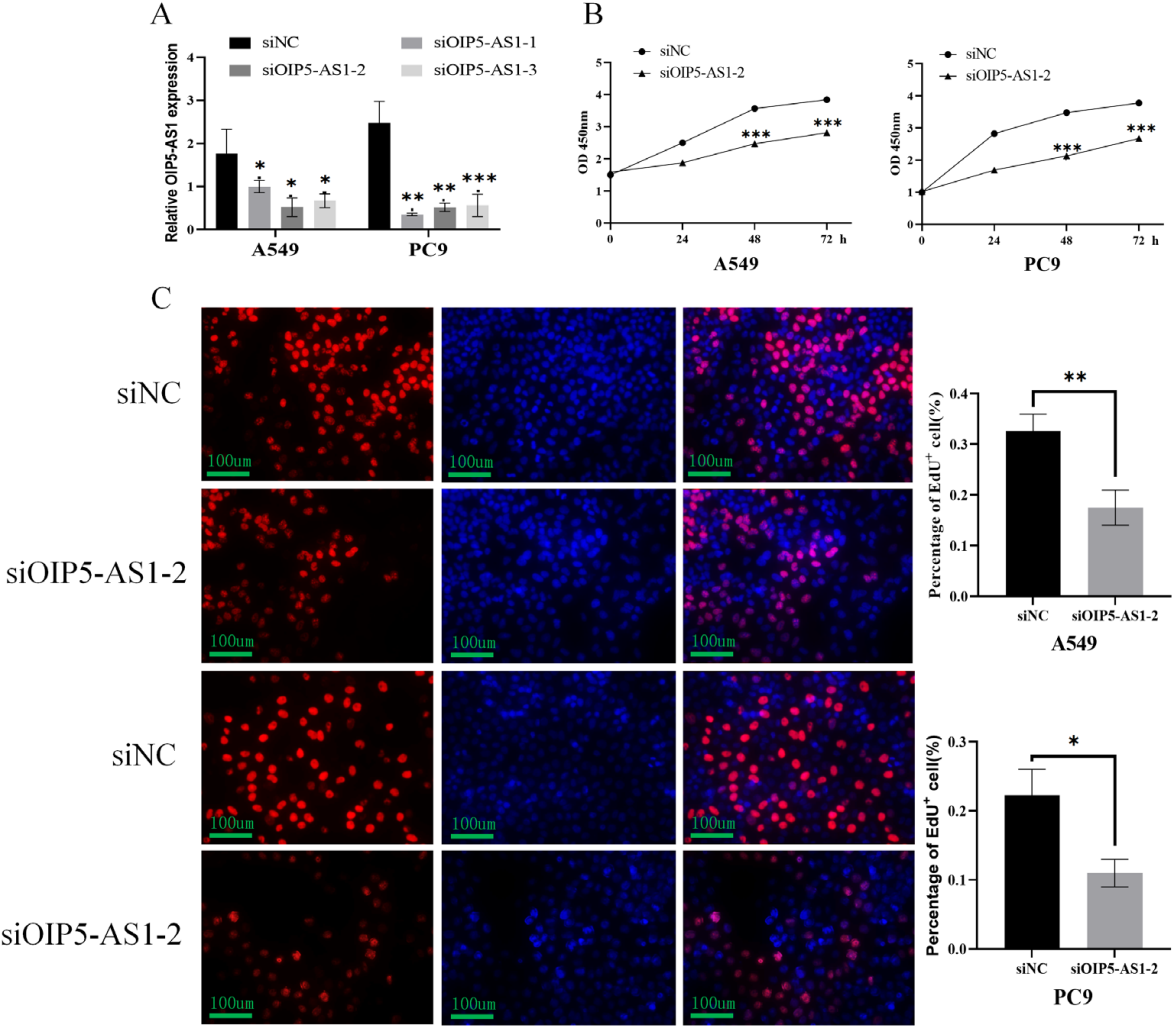
Impact of lncRNA OIP5‐AS1 on the growth and proliferation of lung cancer cell lines A549 and PC9. (A) OIP5‐AS1 expression was quantified by qPCR in A549 and PC9 cells transfected with si‐OIP5‐AS1 sequences. (B) CCK‐8 assays measured cell viability in A549 and PC9 cells transfected with siOIP5‐AS1‐2 or siNC. (C) EdU assays were done to see how OIP5‐AS1‐2 affects A549 and PC9 cell proliferation, counting EdU‐positive cells as a proliferation indicator. Three independent experiments were performed. Error bars stand for the mean ± SD of at least triplicate experiments. *p < 0.05; **p < 0.01; ***p < 0.001.

**FIGURE 3:**
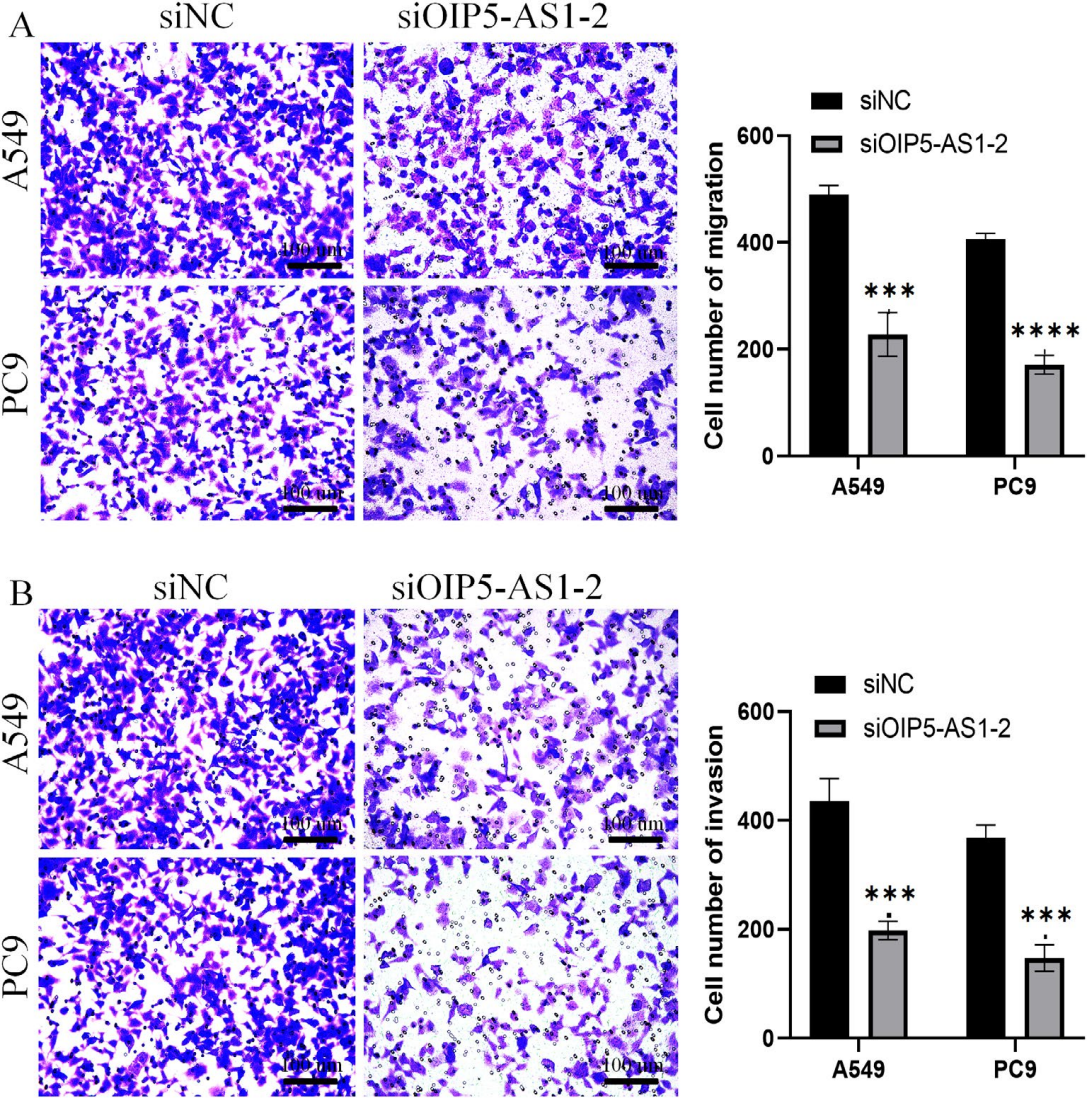
Regulation of lncRNA OIP5‐AS1 on the migration and invasion of A549 and PC9 cells. Migration (A) and invasion (B) assays were conducted to assess the number of A549 and PC9 cells that migrated and invaded, respectively, following transfection with siOIP5‐AS1‐2. Three independent experiments were performed. Error bars stand for the mean ± SD of at least triplicate experiments. ***p < 0.001,****p < 0.0001.

To specifically inhibit OIP5‐AS1 expression, we utilised A549 and PC9 cellular models and delivered three unique siRNA sequences: siOIP5‐AS1‐1, siOIP5‐AS1‐2 and siOIP5‐AS1‐3, designed for maximal knockdown efficiency. Relative to controls, qRT‐PCR measurements confirmed significant OIP5‐AS1 transcript reduction in siRNA‐transfected A549 and PC9 cells (*p* < 0.05, *p* < 0.01, Figure 2A). Compared to negative control siRNA, siOIP5‐AS1‐2 transfection substantially attenuated proliferation rates in A549 and PC9 cells as measured by CCK‐8 assay (*p* < 0.001, Figure 2B). Furthermore, the Edu incorporation assay indicated a reduction in Edu‐positive cells by 46.3% in A549 and 50.7% in PC9 following OIP5‐AS1 knockdown (*p* < 0.05, Figure 2C). As depicted in Figure 3A, loss of function of OIP5‐AS1 resulted in a pronounced inhibition of migratory phenotypes in both NSCLC cell lines (A549 and PC9; *p* < 0.01 for both). Similarly, down‐regulating OIP5‐AS1 led to a significant decrease in cell invasion in A549 (*p* < 0.01) and PC9 cells (*p* < 0.001, Figure 3B). These findings collectively suggest that OIP5‐AS1 knockdown substantially inhibits LUAD cell proliferation, migration and invasion.

### 3.3 hsa‐miR‐29b‐3p Regulated LUAD Cell Behaviour via OIP5‐AS1 3′UTR Targeting

**FIGURE 4:**
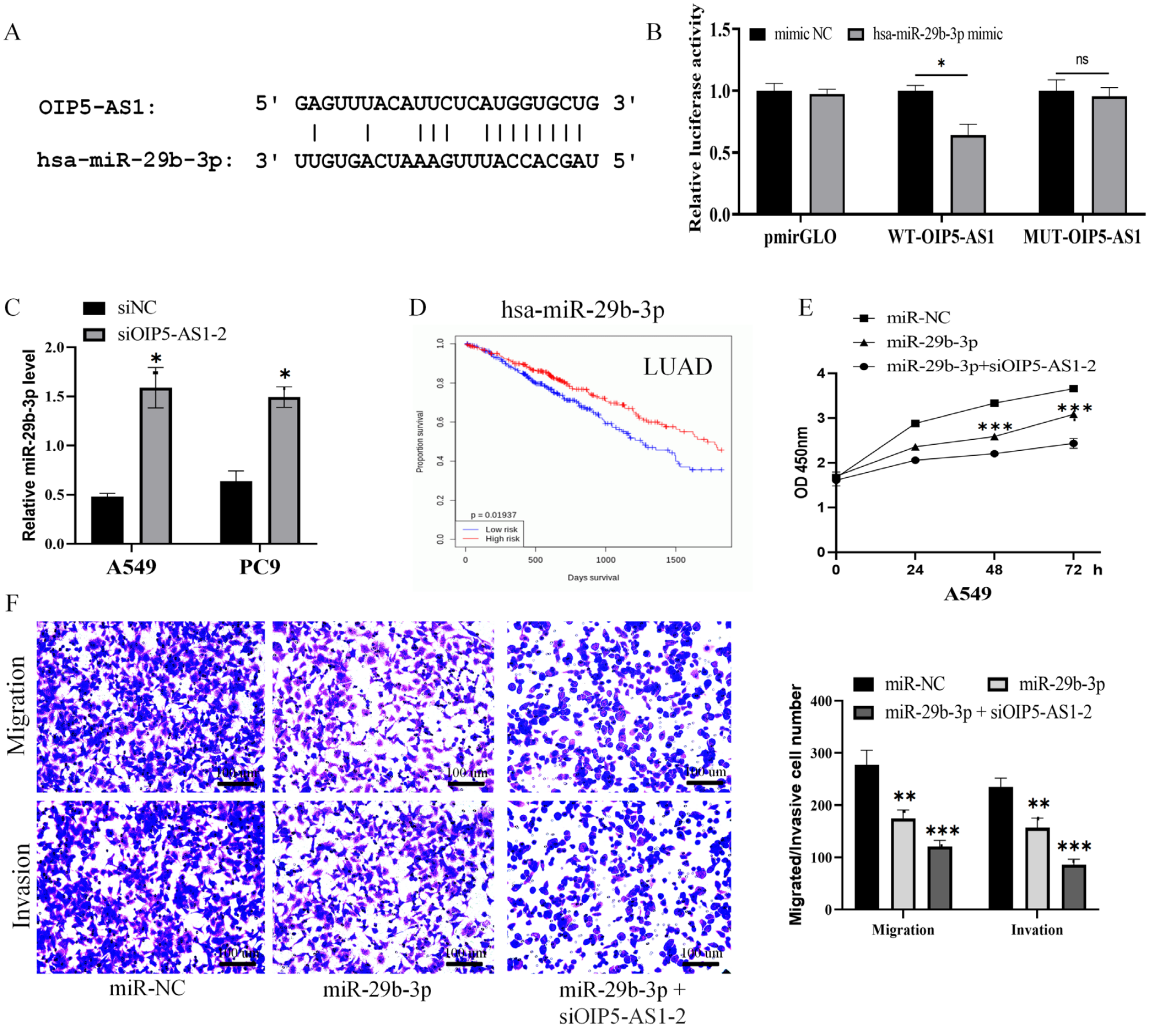
hsa‐miR‐29b‐3p is a direct target of lncRNA OIP5‐AS1. (A) StarBase predicted miRNAs regulated by OIP5‐AS1, revealing a binding site with hsa‐miR‐29b‐3p. (B) Luciferase activity was measured in constructs with WT or MUT OIP5‐AS1 sequences, stimulated with miR‐NC or miR‐29b‐3p. (C) A549 and PC9 cells were transfected with siOIP5‐AS1‐2 or siNC, respectively. miR‐29b‐3p expression was detected by real‐time quantitative PCR after 48 h. (D) Kaplan–Meier analysis using the OncomiR platform showed miR‐29b‐3p significantly impacts LUAD patient survival. (E) CCK‐8 assays were conducted to assess cell proliferation, and (F) transwell assays were employed to evaluate migration and invasion in A549 cells transfected with miR‐NC, miR‐29b‐3p, or co‐transfected with miR‐29b‐3p and siOIP5‐AS1‐2. Three independent experiments were performed. Error bars stand for the mean ± SD of at least triplicate experiments. *p < 0.05; **p < 0.01; ***p < 0.001. LUAD, lung adenocarcinoma.

Evidence suggests that lncRNAs can regulate miRNAs by acting as sponges for them. Employing the Starbase online resource, we detected the presence of complementary binding sites between hsa‐miR‐29b‐3p and the 3′‐UTR of OIP5‐AS1 (Figure 4A). To validate this interaction, a dual‐luciferase reporter assay was performed. Luciferase assays revealed that hsa‐miR‐29b‐3p inhibited WT‐OIP5‐AS1 activity, with a reduction rate of 35.64% (*p* < 0.05, Figure 4B), whereas no significant effect was observed in MUT‐OIP5‐AS1. A notable upregulation in hsa‐miR‐29b‐3p expression was detected by qPCR in both A549 and PC9 cells following OIP5‐AS1 knockdown compared to control groups (*p* < 0.05, Figure 4C). Using the Kaplan–Meier Plotter tool on the OncomiR platform, we evaluated the prognostic significance of hsa‐miR‐29b‐3p expression. Our data associate elevated hsa‐miR‐29b‐3p levels with improved OS in LUAD patients (*p* = 0.01937, Figure 4D). Furthermore, we assessed whether OIP5‐AS1/hsa‐miR‐29b‐3p interaction modulates the acquisition of aggressive cellular phenotypes in LUAD models. Overexpression of hsa‐miR‐29b‐3p significantly inhibited cell proliferation (*p* < 0.001, Figure 4E) and reduced both cell migration and invasion (*p* < 0.01, Figure 4F). These effects were further amplified by the knockdown of OIP5‐AS1 in A549 cells.

### 3.4 OIP5‐AS1 Positively Regulated ZIC5 Expression via hsa‐miR‐29b‐3p

**FIGURE 5:**
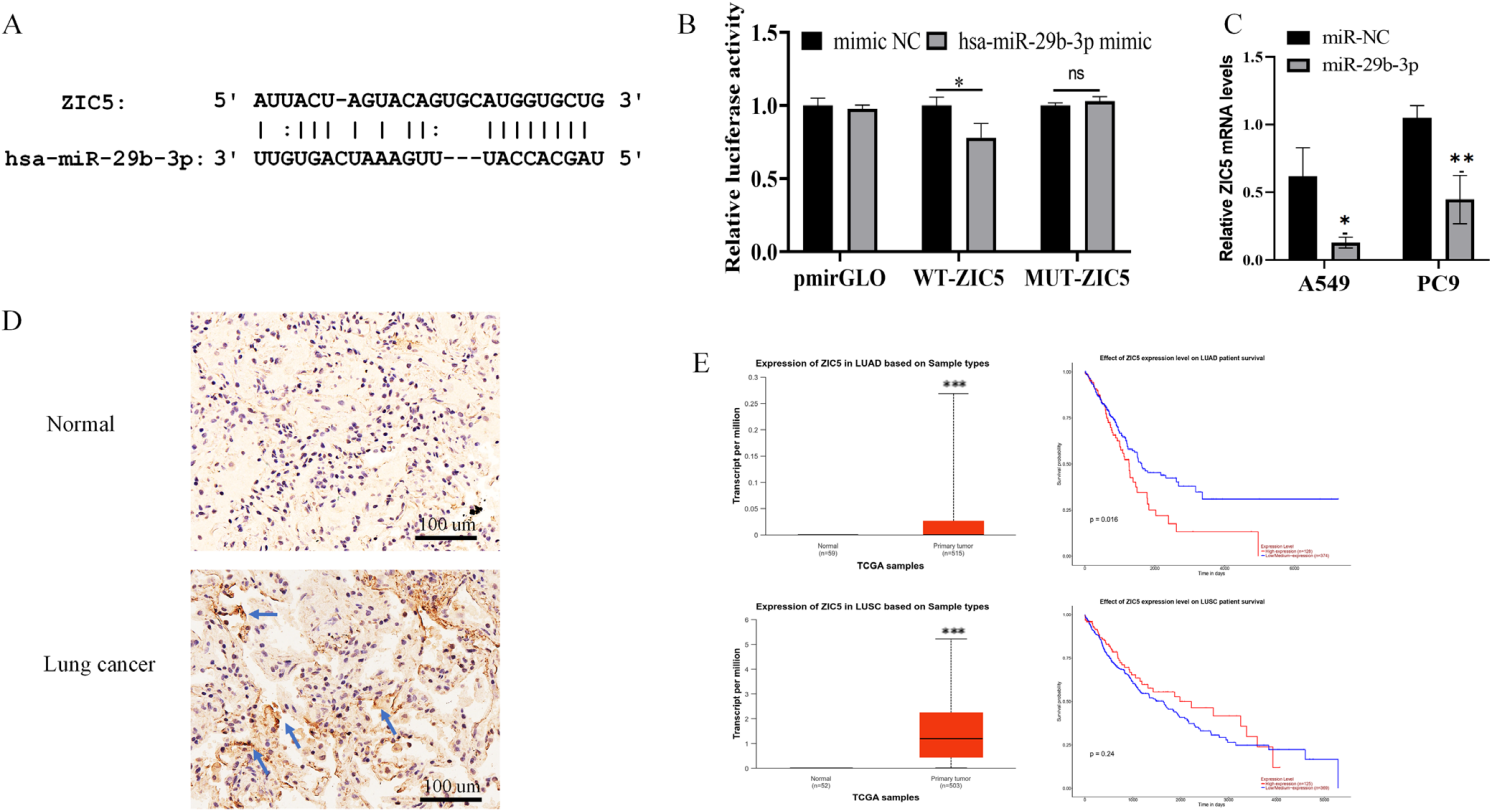
LncRNA OIP5‐AS1 positively regulated ZIC5 expression via miR‐29b‐3p. (A) The starBase online site predicts the interaction site between miR‐29b‐3p and ZIC5. (B) Wild‐type/mutant ZIC5 luciferase reporter vectors were built, and luciferase activity was detected after adding miR‐29b‐3p or miR‐NC. (C) qPCR was used to assess ZIC5 expression in A549 and PC9 cells. (D) Immunohistochemical analysis of ZIC5 expression differences between human normal lung and LUAD tissues. The blue arrow marks the location of ZIC5 expression; 200×. (E) UALCAN database was used to investigate ZIC5 expression and its impact on survival in LUAD and LUSC patients. Three independent experiments were performed. Error bars stand for the mean ± SD of at least triplicate experiments. *p < 0.05; **p < 0.01; ***p < 0.001. LUAD, lung adenocarcinoma; LUSC, lung squamous cell carcinoma.

Through computational target prediction analysis, we discovered a complementary sequence match between hsa‐miR‐29b‐3p and the ZIC5 3′‐UTR (Figure 5A). Functional validation through luciferase reporter assays demonstrated that hsa‐miR‐29b‐3p significantly suppressed WT‐ZIC5 activity by 22.06% (*p* < 0.05, Figure 5B). Additionally, qPCR results demonstrated that elevated levels of hsa‐miR‐29b‐3p led to a decline in ZIC5 mRNA in both A549 and PC9 cell lines (*p* < 0.05; *p* < 0.01, Figure 5C). Immunohistochemical analysis has validated that the level of ZIC5 expression is notably elevated in lung cancer tissues when contrasted with normal tissues (Figure 5D). We utilised the UALCAN platform to examine the transcription levels of ZIC5 in both LUAD and LUSC cohorts and investigated its potential impact on OS in cases involving these cancer types. The analysis revealed that ZIC5 was significantly overexpressed in both the LUAD cohort (tumour = 515 cases, normal = 59 cases, *p* < 0.001) and the LUSC cohort (tumour = 503 cases, normal = 52 cases, *p* < 0.001). The Kaplan–Meier survival analysis revealed that individuals within the LUAD cohort exhibiting high levels of ZIC5 expression had a notably shorter OS (high expression = 128 cases, low/medium expression = 374 cases; *p* = 0.016). Nevertheless, within the LUSC cohort, there was no discernible difference in OS between patients with elevated ZIC5 expression and those with lower or moderate expression levels (high expression = 125 cases, low/medium expression = 369 cases; *p* = 0.24), as shown in Figure 5E. Our findings indicate that heightened ZIC5 expression could be correlated with poor survival prospects in LUAD cases, whereas in LUSC, ZIC5 does not seem to significantly influence OS.

### 3.5 OIP5‐AS1 Promoted Cancer In Vivo Partly by Suppressing hsa‐miR‐29b‐3p

**FIGURE 6:**
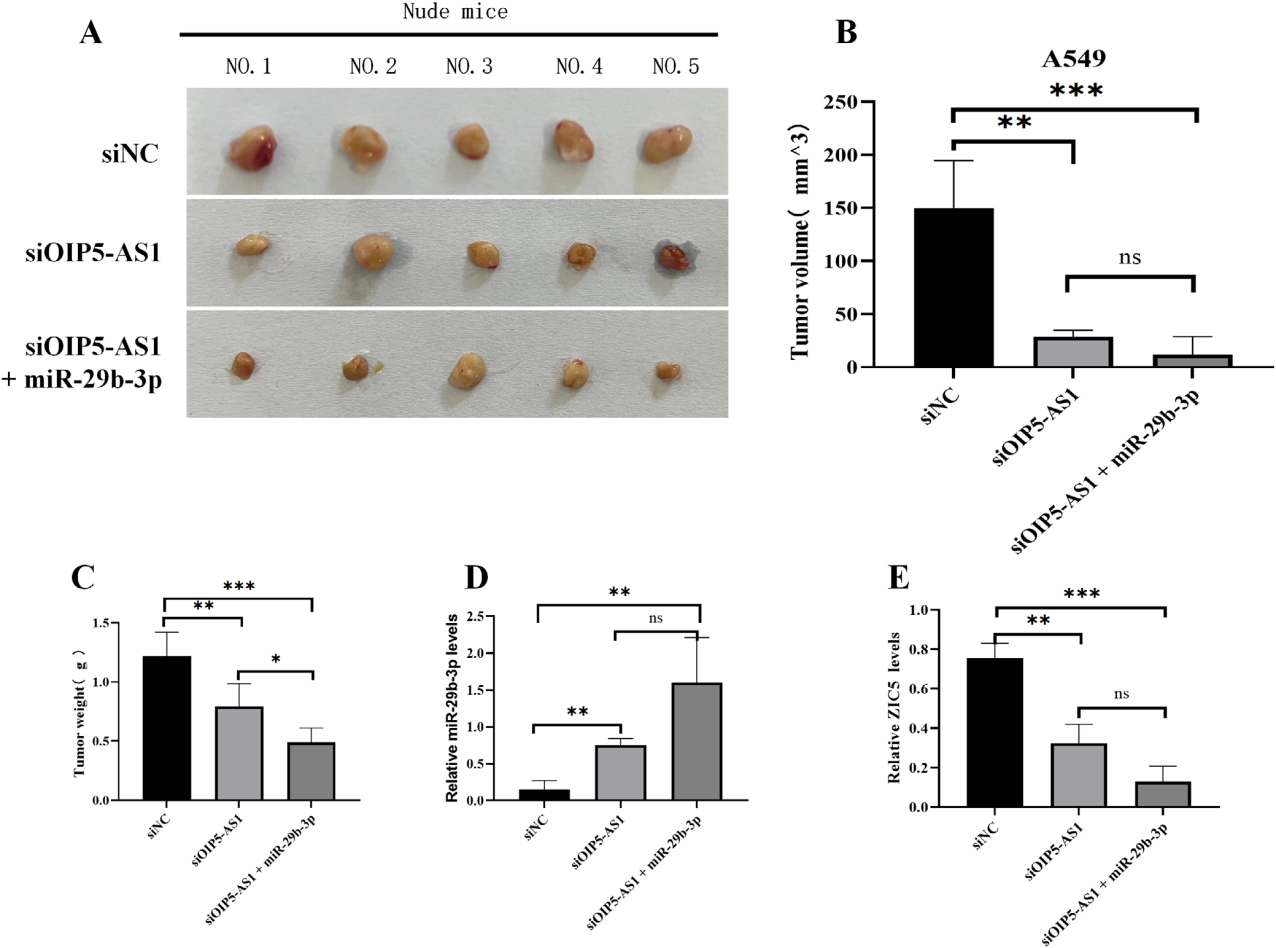
The oncogenic activity of lncRNA OIP5‐AS1 is partly mediated by the negative regulation of miR‐29b‐3p in vivo. (A) The diagram outlines comparative xenograft tumour development in nude mice across three distinct groups: Baseline siNC control (*N* = 5 mice), siOIP5‐AS1‐2 experimental (*N* = 5 mice) and combined miR‐29b‐3p + siOIP5‐AS1‐2 intervention (*N* = 5 mice) cohorts. (B) Tumour volumes were measured in nude mice from three groups: SiNC, siOIP5‐AS1‐2 and miR‐29b‐3p + siOIP5‐AS1‐2. (C) Tumour weight measurements were documented across three nude mice cohorts: SiNC control, siOIP5‐AS1‐2 intervention and combined miR‐29b‐3p + siOIP5‐AS1‐2 treatment groups, each containing five mice. (D) Expression levels of miR‐29b‐3p in xenograft tumours. (E) mRNA expression of ZIC5 in xenograft tumours. At least three independent experiments were performed. Error bars stand for the mean ± SD of at least triplicate experiments. *p < 0.05; **p < 0.01; ***p < 0.001.

To substantiate our cell‐based findings, we implemented a subcutaneous xenograft model using nude mice. The mice were assigned to three groups: siNC, siOIP5‐AS1‐2 and hsa‐miR‐29b‐3p + siOIP5‐AS1‐2. As shown in Figure 6A, significant differences in tumour formation were observed among the groups. Morphometric analysis using diameter measurements and volume extrapolation showed marked reductions in tumour volumes for the siOIP5‐AS1‐2 and combination therapy groups (*p* < 0.01, Figure 6B). Similarly, changes in tumour weight followed the same trend as tumour volume (*p* < 0.01, Figure 6C). We also conducted qRT‐PCR analyses to measure the abundance of hsa‐miR‐29b‐3p and ZIC5 transcripts in the xenograft tumours. In line with the in vitro findings, OIP5‐AS1 knockdown significantly increased hsa‐miR‐29b‐3p expression (*p* < 0.01, Figure 6D) and decreased ZIC5 expression (*p* < 0.01, Figure 6E). The combined treatment of OIP5‐AS1 knockdown and hsa‐miR‐29b‐3p overexpression not only amplified the above effect but also had a profound impact on the overall biological processes being studied, potentially altering the course of the disease or cellular response (*p* < 0.001).

## 4 Discussion

The involvement of lncRNA in cancer pathogenesis is underscored by their dysregulated expression patterns across multiple malignancies, including lung [11], breast [12, 13] and colon [14]. Among these, OIP5‐AS1 has emerged as a pivotal player in regulating tumour growth and metastasis. While prior investigations have implicated OIP5‐AS1 in diverse oncogenic contexts, notably lung carcinoma, the molecular pathways governing its functional contributions remain poorly characterised. Our combined bioinformatics and experimental validation revealed OIP5‐AS1 promotes LUAD advancement by regulating the previously unknown hsa‐miR‐29b‐3p/ZIC5 oncogenic pathway.

Our investigation underscores the notable upregulation of OIP5‐AS1 in LUAD, hinting at its possible part as a marker for the advancement of the disease. Our analysis revealed a pronounced association between OIP5‐AS1 overexpression and critical clinical indicators, particularly tumour dimensions, TNM staging progression and distant metastatic dissemination. These associations indicate that OIP5‐AS1 might play an important role in the aggressive traits typically seen in lung cancer. To reinforce these discoveries, our laboratory and animal studies demonstrated that inhibiting OIP5‐AS1 markedly reduces the growth, motility and invasive properties of LUAD cells. The observed inhibitory effect underscores the oncogenic contribution of OIP5‐AS1 and implicates it as a promising candidate for targeted molecular therapies.

LncRNAs perform multifaceted biological roles that are tightly linked with microRNA (miRNA) activity, forming regulatory networks that critically influence tumour development [15, 16, 17]. OIP5‐AS1 is reported in the literature to be involved in the regulation of cancer. Work by Mei's team [18] identified HDAC7‐mediated epigenetic control as a pathway through which OIP5‐AS1 influences NSCLC metastasis. Supporting evidence from Jiang et al. [19] demonstrated that this lncRNA functions as a miRNA sponge, effectively suppressing the regulatory activity of miR‐142‐5p in lung cancer cells. Previous studies have confirmed the oncogenic role of OIP5‐AS1 in lung cancer development, consistent with our results. However, comprehensive investigations into its regulatory networks and molecular mechanisms controlling cell proliferation and invasion remain insufficient. Through systematic luciferase reporter analysis, we identified critical binding regions between OIP5‐AS1 and hsa‐miR‐29b‐3p. Our data positions hsa‐miR‐29b‐3p as a master regulator of OIP5‐AS1's oncogenic potential in LUAD, offering a tractable therapeutic target for disrupting the lncRNA‐mediated tumour survival network. Cellular analysis showed that increased hsa‐miR‐29b‐3p reduced A549 cell invasion, similar to OIP5‐AS1 downregulation. Interestingly, the overexpression of hsa‐miR‐29b‐3p further enhanced the effects of OIP5‐AS1 knockdown on the behaviour of LUAD cells. Beyond its established role in lung malignancies, research conducted by Zizheng Wu [20] and colleagues demonstrated that OIP5‐AS1 is aberrantly overexpressed in breast cancer specimens. Molecular analysis identifies a novel RNA‐mediated mechanism of breast cancer pathogenesis, characterised by lncRNA‐dependent suppression of miR‐216a‐5p and subsequent GLO1 dysregulation. LncRNAs operate as endogenous molecular sponges for miRNAs in cancer, playing a critical role in regulating downstream gene expression [21]. Consistent with its role as a miRNA sponge, OIP5‐AS1 exhibits binding capacity for various miRNAs such as miR‐338‐3p, miR‐448 and miR‐378a‐3p^6^. Here we provide novel evidence that this lncRNA mediates suppressive effects on hsa‐miR‐29b‐3p via complementary base‐pairing interactions. Previous research has emphasised the crucial role of hsa‐miR‐29b‐3p in various cancers. Liao [22] noted that circHYBID might regulate hyaluronic acid metabolism and promote osteoarthritis through the hsa‐miR‐29b‐3p/TGF‐β1 axis. Peng Song et al. [9] found that LIFR‐AS1 binds with hsa‐miR‐29b‐3p, reducing colon cancer cell invasion and colony formation. Utkarsh Bhardwaj [23] confirmed that hsa‐miR‐29b‐3p affects adhesion junction protein expression in brain microvascular endothelial cells. Thus, we speculate that hsa‐miR‐29b‐3p is closely linked to cell adhesion functions, contributing significantly to tumour progression.

The cerebellar zinc finger (ZIC) gene family comprises five members (ZIC1‐5), whose protein products are defined by characteristic zinc finger motifs. These structurally distinct transcription factors play essential roles across diverse biological processes, particularly in mediating DNA binding and orchestrating complex gene expression networks. Recent investigations reveal the oncogenic role of ZIC5 in pulmonary and hepatic malignancies, where it exerts pro‐tumorigenic effects via transcriptional activation of the CCNB1/CDK1 complex and stabilisation of β‐catenin‐mediated signalling [24, 25]. Reiko's findings [26] suggest that the overexpression of ZIC5 can enhance the invasiveness of melanoma primarily by activating the FAK and STAT3 signalling pathways. Study findings indicate a distinct upregulation of ZIC5 in LUAD relative to LUSC, with adenocarcinoma cases presenting significantly increased ZIC5 expression that specifically associates with adverse survival probabilities. Utilising rigorous computational analyses, we have validated ZIC5 as a functionally relevant target of hsa‐miR‐29b‐3p in the LUAD microenvironment. In vivo studies revealed a direct causal link between OIP5‐AS1 suppression and the upregulation of hsa‐miR‐29b‐3p, which subsequently led to the downregulation of ZIC5 expression. This regulatory axis significantly impaired the oncogenic properties of LUAD cells, particularly their proliferative and invasive capacities. These results suggest that OIP5‐AS1 may act as an oncogene in the development of LUAD. Our findings revealed reciprocal relationships between OIP5‐AS1 and hsa‐miR‐29b‐3p, as well as between hsa‐miR‐29b‐3p and ZIC5. The identified interaction between OIP5‐AS1 and the hsa‐miR‐29b‐3p/ZIC5 regulatory module represents a critical node in the LUAD molecular network, offering new therapeutic entry points for systemic intervention.

While this study presents compelling evidence regarding the oncogenic role of OIP5‐AS1 in LUAD, several limitations should be considered. First, a primary limitation lies in the experimental models employed, particularly A549 and PC9 cell lines and xenograft systems, which may inadequately recapitulate the intricate clinical complexity and tumour heterogeneity inherent to LUAD. Differences in the tumour microenvironment and genetic background between cell lines and actual patient tumours could limit the direct clinical relevance of the findings. Second, the generalisability of the results to different LUAD subtypes remains uncertain. Given the remarkable genetic and molecular heterogeneity of LUAD, the functional uniformity of the OIP5‐AS1/hsa‐miR‐29b‐3p/ZIC5 axis across distinct molecular subtypes remains to be elucidated.

In summary, our research provides persuasive evidence that OIP5‐AS1 operates as a pivotal oncogenic factor in lung cancer, markedly enhancing cellular proliferation and invasive capabilities. Mechanistically, OIP5‐AS1 contributes to tumorigenesis via its modulation of the hsa‐miR‐29b‐3p/ZIC5 axis, revealing a sophisticated regulatory circuitry that governs critical aspects of tumour biology. Deciphering the molecular mechanisms governing these pathways advances our knowledge of lung cancer and creates foundational opportunities for designing innovative targeted therapies.

## Author Contributions

**Long Liang:** writing – original draft (equal), writing – review and editing (equal). **Ying Luo:** validation (equal), visualization (equal). **Danyang Li:** data curation (equal), formal analysis (equal). **Yongchang Sun:** conceptualization (equal), writing – review and editing (equal).

## Ethics Statement

This study adhered to ethical standards and Helsinki principles, approved by Peking University Third Hospital's Ethics Committee (No. S2024204). Animal experiments were also approved under the same number, with all procedures following relevant guidelines.

## Conflicts of Interest

The authors declare no conflicts of interest.

## Supporting information


Appendix S1.

## Data Availability

The data presented in the article is available from online databases.
