# Supplementary material for: LncRNA OIP5‐AS1 Modulates the Biological Behaviour of Lung Cancer Cells by Regulating the hsa‐miR‐29b‐3p/ZIC5 Axis
Source: J Cell Mol Med. 2025 May 8;29(9):e70596. doi: 10.1111/jcmm.70596 (PMC12061636; doi:10.1111/jcmm.70596)
Supplement: Supplementary file 1 — Appendix S1. [file JCMM-29-e70596-s001.docx]

**Table S1**. Gene primer sequence design.

| Human Gene | Forward (5’ – 3’) | Reverse (5’ – 3’) |
| --- | --- | --- |
| OIP5-AS1 | GACCGTGCGAGGAGAAGAAA | CGCGCCTAACACTTTGCTTT |
| hsa-miR-29b-3p | GCGCGTAATACTGCCTGGTAA | AGTGCAGGGTCCGAGGTATT |
| ZIC5 | AAACTTTCGGCACCATGCAC | CGGGGACTTGCAGTGAATCT |
| GAPDH | TCCAAAATCAAGTGGGGCGA | AAATGAGCCCCAGCCTTCTC |
| U6 | CTCGCTTCGGCAGCACA | AACGCTTCACGAATTTGCGT |

**Table S2.** siRNA primer sequence design.

| siRNA | Sense (5’ – 3’) | Antisense (5’ – 3’) |
| --- | --- | --- |
| siOIP5-AS1-1 | CAAACAGGCUUUGUGUUCCUUAUCA dTdT | UGAUAAGGAACACAAAGCCUGUUUG dAdT |
| siOIP5-AS1-2 | CAGAGUAUUGGAACAUCUCUGUGUU dTdT | AACACAGAGAUGUUCCAAUACUCUG dAdT |
| siOIP5-AS1-3 | CAGGUGUCUGUUAAACCCAUCUUCU dTdT | AGAAGAUGGGUUUAACAGACACCUG dAdT |
| siNC | CAAGACGUUGUGUUUCCUUACAUCA dTdT | UGAUGUAAGGAAACACAACGUCUUG dAdT |
